# Profibrillatory Structural and Functional Properties of the Atrial-Pulmonary Junction in the Absence of Remodeling

**DOI:** 10.3389/fphys.2021.748203

**Published:** 2021-11-24

**Authors:** Lisa A. Gottlieb, Charly Belterman, Shirley van Amersfoorth, Virginie Loyer, Marion Constantin, Mélèze Hocini, Lukas R. C. Dekker, Ruben Coronel

**Affiliations:** ^1^Department of Experimental Cardiology, Location Academic Medical Centre, Amsterdam University Medical Centre, Amsterdam, Netherlands; ^2^IHU Liryc, University of Bordeaux, Bordeaux, France; ^3^Department of Biomedical Engineering, Eindhoven University of Technology, Eindhoven, Netherlands; ^4^Department of Cardiology, Catharina Hospital, Eindhoven, Netherlands

**Keywords:** atrial fibrillation, arrhythmogenesis, pulmonary vein, histology, sheep model, refractoriness

## Abstract

**Background**: Sole pulmonary vein (PV) isolation by ablation therapy prevents atrial fibrillation (AF) in patients with short episodes of AF and without comorbidities. Since incomplete PV isolation can be curative, we tested the hypothesis that the PV in the absence of remodeling and comorbidities contains structural and functional properties that are proarrhythmic for AF initiation by reentry.

**Methods:** We performed percutaneous transvenous *in vivo* endocardial electrophysiological studies and quantitative histological analysis of PV from healthy sheep.

**Results:** The proximal PV contained more myocytes than the distal PV and a higher percentage of collagen and fat tissue relative to myocytes than the left atrium. Local fractionated electrograms occurred in both the distal and proximal PVs, but a large local activation (>0.75 mV) was more often present in the proximal PV than in the distal PV (86 vs. 50% of electrograms, respectively, *p* = 0.017). Atrial arrhythmias (run of premature atrial complexes) occurred more often following the premature stimulation in the proximal PV than in the distal PV (*p* = 0.004). The diastolic stimulation threshold was higher in the proximal PV than in the distal PV (0.7 [0.3] vs. 0.4 [0.2] mA, (median [interquartile range]), *p* = 0.004). The refractory period was shorter in the proximal PV than in the distal PV (170 [50] vs. 248 [52] ms, *p* < 0.001). A linear relation existed between the gradient in refractoriness (distal-proximal) and atrial arrhythmia inducibility in the proximal PV.

**Conclusion:** The structural and functional properties of the native atrial-PV junction differ from those of the distal PV. Atrial arrhythmias in the absence of arrhythmia-induced remodeling are caused by reentry in the atrial-PV junction. Ablative treatment of early paroxysmal AF, rather than complete isolation of focal arrhythmia, may be limited to inhibition of reentry.

## Introduction

Atrial fibrillation (AF) is a common cardiac arrhythmia in humans, which decreases the quality of life and increases the risk of ischemic stroke and heart failure (Jenkins and Bubien, 1996; Kannel and Benjamin, 2009). Premature atrial complexes (PAC) originating from the pulmonary vein (PV) myocardial sleeve are considered to trigger paroxysms of AF (Haissaguerre et al., 1998). The PV ectopy may be caused by abnormal automaticity, triggered activity, or reentry (Hocini et al., 2002).

Since a focal mechanism for PV ectopy is often suggested, drug-resistant paroxysmal AF (duration of AF episode < 1 week) has been treated with ablative therapy using “spot-on” targeted PV ablation (Haissaguerre et al., 1998) or complete PV isolation (PVI) lesions (Pappone et al., 1999). In PVI, ablation lesions are created to prevent ectopy from the PV from activating the left atrium (LA) (Calkins et al., 2018). The highest success rate of sole PVI exists in patients with short episodes of AF (paroxysmal AF), with low extent of structural fibrotic AF remodeling, and without comorbidities (Themistoclakis et al., 2008; Marrouche et al., 2014; Kis et al., 2017).

Incomplete PVI (defined as persisting electrical reconnection between the PV and LA) can be curative of paroxysmal AF (Cappato et al., 2003; Verma et al., 2005; Pratola et al., 2008; Jiang et al., 2014; Kuck et al., 2016). Moreover, the ablation success is independent of the procedural endpoints of electrical isolation (Stabile et al., 2003; Lemola et al., 2005; Pratola et al., 2008). These observations suggest that, at least in a subset of patients with paroxysmal AF without structural remodeling and comorbidities, arrhythmogenic mechanisms in the PV underlying the initiation of AF are amenable to ablation strategies other than complete PVI. We, therefore, hypothesized that the structural and functional properties of the native PV myocardium in the absence of comorbidities facilitate the initiation of PAC by reentrant activation.

We provided the trigger by programmed stimulation protocols to investigate the arrhythmogenicity of the localized PV myocardium. We observed that the PV myocardial sleeve in adult sheep, even in the absence of AF-remodeling, constitutes profibrillatory properties conducive to reentry. Our results can have implications for the strategy of ablative therapy in patients with early paroxysmal AF and without comorbidities.

## Materials and Methods

This study was carried out in accordance with the EU Directive 2010/63/EU for protection of animals used for scientific purposes and approved by the local ethical authorities at University of Bordeaux, France (approval number 7995).

### Catheterization

**FIGURE 1:**
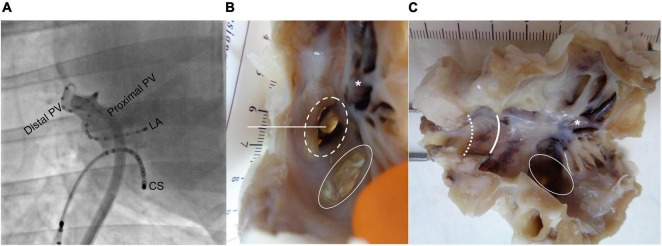
Electrode position and histological preparation. **(A)** Fluoroscopic image (right anterior oblique view 42°) showing the 20-electrode diagnostic spiral catheter positioned in the right pulmonary vein (RPV). A linear catheter is placed in the coronary sinus (CS) as an anatomical reference during the procedure. **(B)** Image of left atrium (LA) with pulmonary vein (PV) before dissection. Dotted circle: RPV. Intact circle: common left pulmonary vein (LPV). Straight line: incision line through the RPV generating **(C)**. Asterisk: trabeculated LA tissue. Intact curved line: transition from LA to proximal RPV. Dotted curved line: transition from proximal to distal RPV.

Fourteen healthy female sheep (2–3 years old; 51.7 ± 6.5 kg; sheep strain: Charmoise) underwent percutaneous transvenous endocardial catheterization under general anesthesia (premedication: 20 mg/kg ketamine + 0.1 mg/kg acepromazine, induction: 1 mg/kg propofol, maintenance: 2% isoflurane). A transseptal puncture was achieved with a steerable sheath under fluoroscopic guidance (Siemens, Erlangen, Germany) and followed by heparin administration (1 mg/kg). A 20-electrode spiral-formed diagnostic catheter (Inquiry AFocusII, St. Jude Medical, Saint Paul, MN, United States) was positioned in the right PV (RPV) in such a manner that the electrodes were in contact with both the proximal and distal PVs (Figure 1A). A reference electrode was placed in the mouth of the sheep.

### Stimulation Protocol

Unipolar electrograms were recorded and stored in Labsystem Pro (BARD EP, Boston Scientific, Marlborough, MA, United States; 4 kHz sampling frequency; filtering: low cutoff 0.05 Hz, high cutoff 500 Hz, adaptive notch filter) during sinus rhythm and during bipolar pacing in the proximal and distal RPVs (Micropace, Santa Ana, CA, United States). The diastolic stimulation threshold was measured decrementally, and pacing was continued with 2 × threshold current. Programmed S1-S2 stimulation with a single premature beat (8 × S1 of 500 ms cycle length followed by 1 × S2 with decreasing coupling interval; 10 ms steps until first loss of local activation where after a 5 ms step continued by 1 ms steps; a pause of 1,000 ms was inserted after each S2) was performed in the proximal and distal RPVs until local loss of activation. Refractory period was defined as the shortest S2 coupling interval causing local activation.

### Histology

Four hearts were explanted following euthanasia by induction of ventricular fibrillation, as approved by the ethical authorities. The RPV and left PV (LPV) were dissected (Figures 1B,C). Of note, the sheep has three PVs, one RPV, and two LPVs. The LPV has a common ostium. A transmural specimen of LA free wall was cut from each sheep, and it served as a control tissue. All specimens were fixated in paraformaldehyde (4%) at 4°C for a minimum of 2 weeks. Dehydration was performed automatically (Leica HistoCore Pearl processor, Wetzlar, Germany) before the specimens were embedded in paraffin. Sections (6 μm thickness) were cut with a microtome; thus, the PV slices included the proximal and distal PVs, while the transmural LA slices contained both the endo- and epicardium. The slices were stained with Masson’s trichrome to visualize myocytes (red), nuclei (black), and collagen (green) before digitally scanned with a 20× objective. The histological images were visualized and sectioned using the Zen Blue software (Zeiss, Oberkochen, Germany).

**FIGURE 2:**
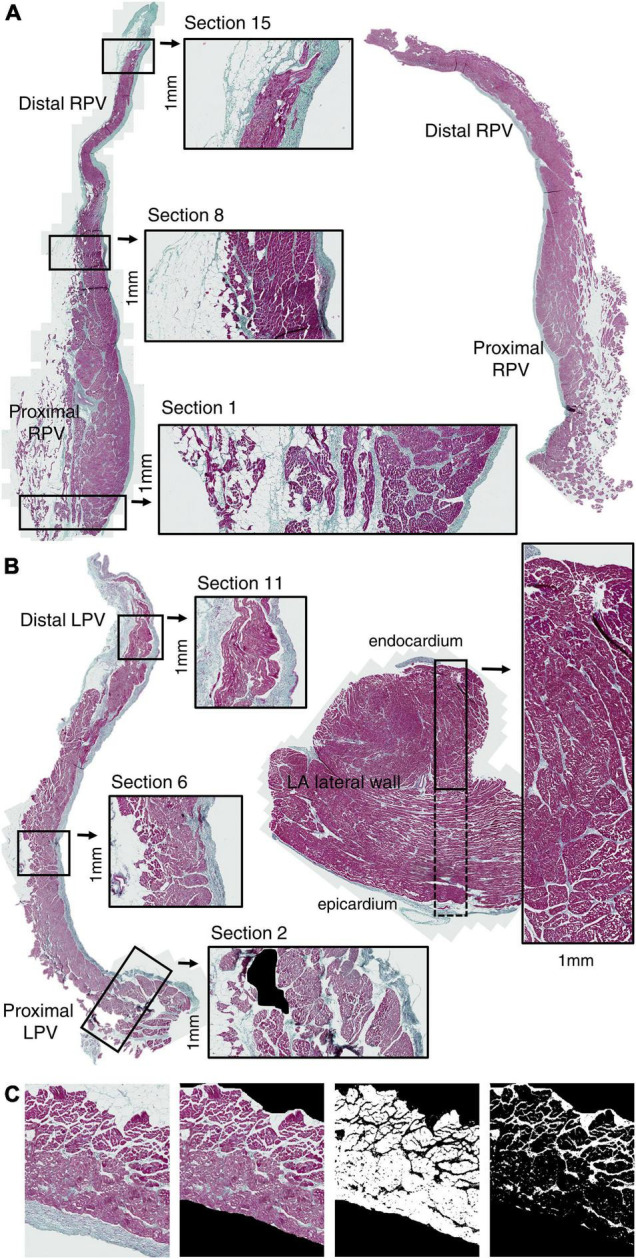
Pulmonary vein histology. Slice along the longitudinal PV axis (proximal-distal) of RPV **(A)** and LPV **(B)** stained with Masson’s trichrome coloration (red: myocytes; green: collagen; black: nuclei). Sections (1 mm width) were positioned along the PV axis from proximal to distal. One transmural (endocardium-epicardium) section (1 mm width) was positioned in each LA lateral wall specimen **(B)**. Quantification of myocyte and collagen-fat tissue was performed in the PV tunica media excluding the endothelium and adventitia **(C)**. Artifacts, vessels, and perivascular collagen were also excluded from the analysis. Note the accumulation of collagen and fat tissue in the proximal PV.

One slice without large areas of tissue separation from each of the PV and LA specimens was chosen for the quantification analysis. Sections of a fixed width of 1 mm were positioned along the PV slices from proximal to distal (Figures 2A,B). A region of interest was manually selected in each section, thus including only the PV tunica media spanning from the first myocyte lateral to the endocardium to the last myocyte medial to the adventitia (Figure 2C). Intramural vessels, perivascular connective tissue, and smaller areas of tissue distortion were excluded from the analysis (Figure 2B). One section encompassing the atrial wall was positioned in each of the four LA slices.

Quantification of myocyte area was done in each region of interest using the ImageJ software (LOCI, University of Wisconsin, WI, United States) by color thresholding and was expressed as mm^2^. Similarly, collagen and fat tissue between the myocytes were quantified in combination by thresholding for white and green areas. We calculated the collagen-fat percentage (area_collagen–fat_/(area_myocyte_ + area_collagen–fat_) × 100) and considered that any tissue distortion (included as white area) was relatively similar in analyzed regions. The total tissue area was area_myocyte_ + area_collagen–fat_.

We reasoned that reentrant activations are more likely to occur in areas with collagen-fat tissue intermingled with myocytes in the presence of a sufficient mass of myocardium (Garrey, 1914; Kawara et al., 2001). According to this concept, a high myocyte content and a relatively high amount of collagen-fat tissue between myocytes present a high risk for arrhythmias. Contrarily, a low myocyte amount poses a lesser risk of reentry.

Values in 5 anatomical groups (4 PV groups going from proximal to distal: 1–4 mm, 5–8 mm, 9–12 mm, and 13–18 mm; and 1 LA group) were pooled. The fourth PV group spanned from 13 to 18 mm in order to contain the most distal part of the PV that slightly varied in length.

### Electrophysiological Analysis

Two electrodes in proximity to the bipolar pacing electrodes were selected in each of the proximal and distal RPV regions based on the fluoroscopic contrast images. The unipolar electrograms (four per sheep: two in the proximal RPV and two in the distal RPV) were manually analyzed using a custom-made software based on MATLAB (MathWorks, Natick, MA, United States). Local fractionated electrograms (LFE) were defined as multiple (>1) deflections with depolarization rate dV/dt < 0.3 V/s and maximum deflection magnitude < 0.75 mV. A deflection with a depolarization rate dV/dt > 0.3 V/s and maximum magnitude >0.75 mV was defined as a bulk (B) component.

Premature atrial complexes following a premature S2 stimulus were quantified. The atrial arrhythmia inducibility (AAI) was defined as the total number of PAC occurring during the last five S2 decrements causing local activation and before reaching the refractory period.

### Statistical Analysis

Normality was tested using Shapiro-Wilk test. Data values were expressed as mean ± standard deviation or median (interquartile range), depending on normality and tested with a paired two-tailed Student’s *t*-test or a Wilcoxon signed-rank test, as appropriate. The AAI was compared with a Wilcoxon signed-rank test. A linear regression model was fitted to the AAI in the proximal PV as a function of the difference in refractoriness (distal-proximal PV). Presence of LFE and LFE+B was tested using a Friedman’s test. A mixed-effects linear model was used for testing the quantitative histological parameters. Tukey correction was applied for multiple testing. Statistical significance was assumed at *p*-values < 0.05.

## Results

### Structure

**FIGURE 3:**
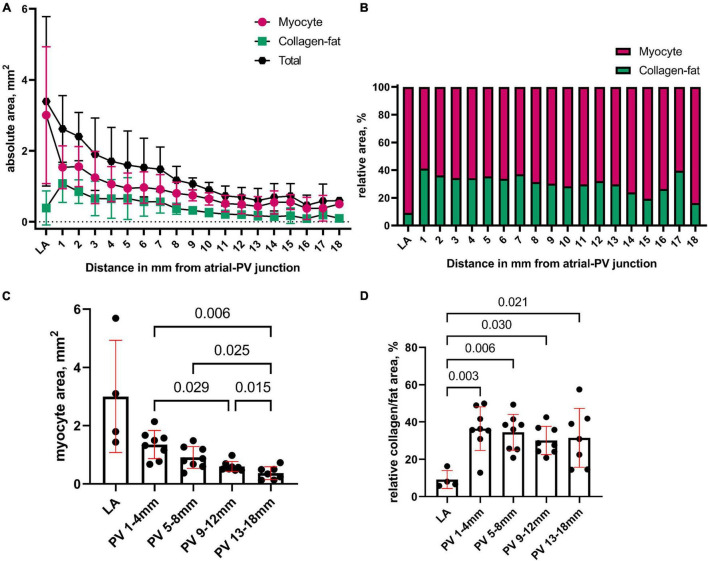
Quantification of the structural composites in the pulmonary vein. **(A)** We quantified the area of myocytes and of collagen-fat tissue (between the myocytes) in the LA and every 1 mm from proximal to distal PV. The total tissue area is area_myocyte_ + area_collagen–fat_. **(B)** Relative percentage of myocytes and of collagen-fat tissue in the LA and PV. **(C)** The myocyte area was pooled in 5 anatomical groups (4 PV groups going from proximal to distal: 1–4 mm, 5–8 mm, 9–12 mm, and 13–18 mm; and 1 LA group). The proximal PV contained significantly more myocytes than the distal PV. **(D)** The relative content of collagen and fat tissue between the myocytes was statistically higher in the PV myocardium than in the LA but did not differ between the proximal and distal PVs. Bars: mean values. Error bars: standard deviation. Mixed-effects linear models were used for the statistical testing, and Tukey correction was applied for multiple testing.

The length of the PV myocardial sleeve from the ostium ranged 13–18 mm and 12–17 mm in the RPV and LPV, respectively. We quantified the area of myocytes and of collagen-fat tissue (between the myocytes) every 1 mm along the length of PV (Figure 3A). Figure 3B shows the relative percentage of myocytes and of collagen-fat tissue in the LA and PV. The proximal PV displayed a significantly larger myocyte area than the distal PV (proximal PV: 1–4 mm (1.35 ± 0.48 mm^2^) vs. distal PV: 13–18 mm (0.37 ± 0.18 mm^2^), *p* = 0.006; Figure 3C), and the myocyte area decreased progressively from the proximal PV to the distal PV.

The PV myocardium contained relatively more collagen-fat tissue than LA, whereas the relative percentage of collagen-fat tissue did not differ between the proximal and distal PV sites (Figure 3D). The myocyte and collagen-fat area did not differ between the RPV and LPV (*p* = 0.882).

Then, we investigated the *in vivo* electrophysiology of the RPV. This PV was chosen due to the higher accessibility in sheep and lack of significant differences in histological composition between the RPV and LPV.

### Local Fractionated Electrograms

**FIGURE 4:**
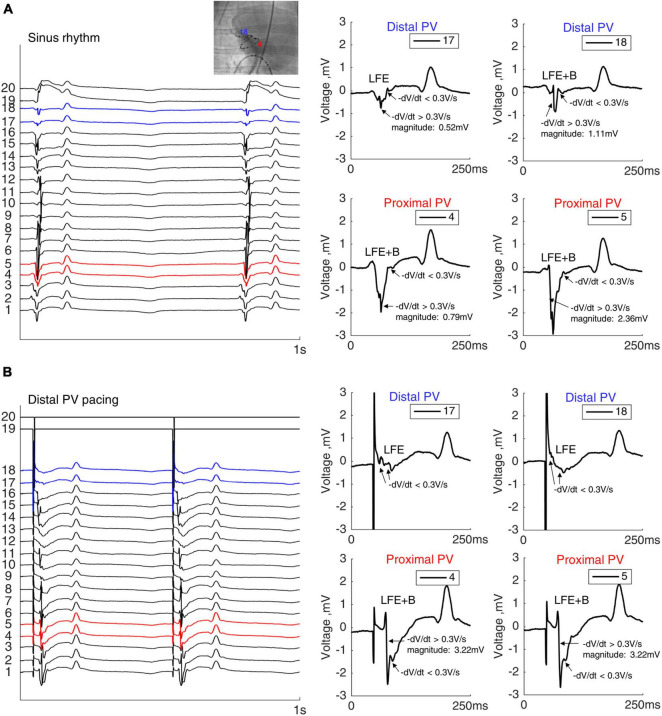
Electrograms in the PV. Twenty unipolar PV electrograms during sinus rhythm **(A)** and distal PV pacing **(B)** in the same sheep. Two electrodes in both distal (blue) and in proximal (red) RPVs were analyzed after verifying the catheter position by fluoroscopy. Local fractionated electrograms with a bulk component (LFE+B) were more frequently observed in the proximal PV than the in distal PV. Note the remote ventricular activation after the PV electrograms. Bipolar stimulation in the distal RPV from electrodes 19 and 20.

We evaluated the morphology of the local electrograms in the distal and proximal PVs during sinus rhythm to investigate a correlation with the histological findings. Figure 4A shows LFE in the distal PV, whereas a larger and steeper deflection (bulk: B) in combination with LFE (LFE+B) was present in the proximal PV. During sinus rhythm, LFE+B was more often observed in the proximal PV than in the distal PV (86 and 50% of total electrograms in the proximal PV (*n* = 28) and the distal PV (*n* = 28), respectively, *p* = 0.017). Similarly, during the distal PV stimulation, LFE+B was more frequent in the proximal PV than in the distal PV (79 vs. 39% of total electrograms, respectively, *p* = 0.007; Figure 4B).

In summary, the rich content of fat and collagen between the myocytes in the PV myocardial sleeve was expressed in the local fractionation in both the distal and proximal PVs, whereas the bulk myocardium in the proximal PV was reflected by the larger deflection (B). The LFE (without B) were predominant in the distal PV due to the absence of bulk myocardium.

### Atrial Arrhythmias in the Proximal Pulmonary Vein

**FIGURE 5:**
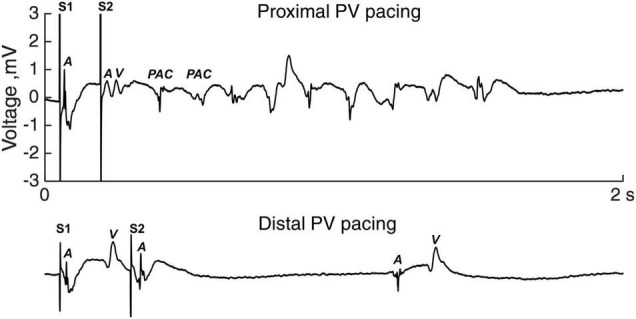
Atrial arrhythmias induction. Electrogram tracings of premature S2 stimulation in the proximal PV (upper panel) and the distal PV (lower panel). The S1-S2 coupling intervals equaled the local refractory period. A, atrial activation; PAC, premature atrial complex; V, ventricular activation.

Figure 5 shows an example of a run of PAC following a short-coupled premature stimulated activation (S2) in the proximal PV (upper trace). Premature stimulation in the distal PV in the same sheep did not induce arrhythmia (Figure 5, lower trace).

**FIGURE 6:**
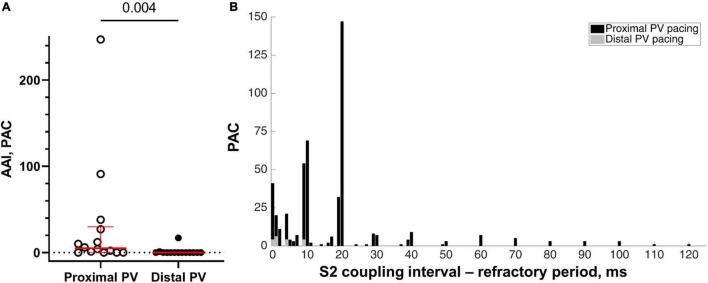
Inducibility of atrial arrhythmias. **(A)** The atrial arrhythmia inducibility was calculated as the sum of PAC during the last five premature S2 decrements of the programmed stimulation protocol causing local activation and before reaching the refractory period (*n* = 14 in the proximal and distal PVs). **(B)** The number of PAC as a function of the difference between the S1-S2 coupling interval and the refractory period. More PAC were induced when the S1-S2 coupling interval approached the refractory period. Note the few PAC during the distal PV pacing (represented in gray color).

Overall, 10 out of 14 sheep demonstrated atrial arrhythmias during the proximal PV pacing (1–147 PAC per run; maximum duration 25 s), whereas 2 out of 14 sheep had atrial arrhythmias during the distal PV pacing (1–6 PAC per run). To overcome the bias of different number of decrements in the S1-S2 protocol, we calculated the AAI. AAI was larger with the proximal PV pacing than with the distal PV pacing (5 [29.8] vs. 0 [0] PAC; proximal vs. distal, *p* = 0.004; Figure 6A).

The incidence of spontaneous PAC increased with S2 coupling intervals closer to the refractory period (Figure 6B). Neither spontaneous atrial arrhythmias nor S1 pacing was observed during sinus rhythm.

### Gradient in Refractoriness

**FIGURE 7:**
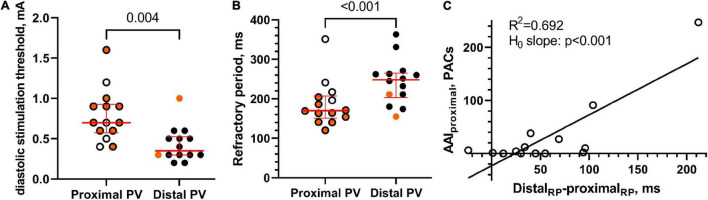
Excitability and refractoriness. **(A)** The diastolic stimulation threshold was higher in the proximal PV than in the distal PV, thereby indicating lower excitability in the proximal PV. Orange color indicates that atrial arrhythmias were induced by local pacing. **(B)** The refractory period was shorter in the proximal PV than in the distal PV. **(C)** A statistically significant linear relation between AAI in the proximal PV and the distal-proximal difference in PV refractory period (RP), *n* = 14.

The diastolic stimulation threshold was higher in the proximal PV than in the distal PV (0.7 [0.3] vs. 0.4 [0.2] mA, respectively, *p* = 0.004; Figure 7A). The refractory period was shorter in the proximal PV than in the distal PV (170 [50] vs. 248 [52] ms, respectively, *p* < 0.001; Figure 7B).

We observed a linear relation between the gradients in PV refractoriness and the AAI in the proximal PV (*R*^2^ = 0.692; slope = 1.0 (95% CI [0.6, 1.4]; hypothesis test of slope equals 0: *p* < 0.001; Figure 7C).

## Discussion

We demonstrated that the native myocardium in the proximal PV facilitates atrial arrhythmias. Atrial arrhythmias were indeed induced following the premature pacing in the proximal PV and significantly less following the pacing in the distal PV. We observed that the proximal PV in the absence of AF remodeling contained more myocytes than the distal PV and relatively more collagen and fat tissue between myocytes than the LA. These observations were reflected in the electrogram morphology by the presence of LFE+B in the proximal PV, whereas the absence of a B (bulk) component reflected the smaller area of myocytes in the distal PV. The number of PAC increased with shorter S2 coupling interval until the refractory period, and a linear relation existed between the local gradient in refractoriness and inducibility of arrhythmia in the proximal PV. This argues for a reentrant mechanism dependent on temporal differences in local activation and repolarization. The local heterogeneity in excitability and the dependence of arrhythmia induction on pacing location support the notion that the atrial arrhythmias were of reentrant origin.

### Structural Pulmonary Vein Properties

The myocardial fiber orientation in the atrial-PV junction is complex with abrupt changes in direction (Saito et al., 2000; Ho et al., 2001; Hocini et al., 2002; Hamabe et al., 2003; Tan et al., 2006). Discontinuities in the myocardium also occur in the atrial-PV junction of human individuals (Hassink et al., 2003). This likely leads to disturbance of the activation wave, thereby facilitating reentrant activation. The structural properties of the atrial-PV junction of healthy sheep resemble that of the normal human heart (Nathan and Eliakim, 1966; Nathan and Gloobe, 1970; Saito et al., 2000; Ho et al., 2001; Tagawa et al., 2001; Hocini et al., 2002; Hamabe et al., 2003; Hassink et al., 2003; Tan et al., 2006).

In addition, collagen and fat tissue embed the myocytes in the PV sleeves from non-AF individuals similar to our observations in healthy sheep (Ho et al., 2001; Kugler et al., 2018). Likewise, collagen and fat tissue disturb the electrical conduction in the myocardium by creating activation heterogeneity, conduction block, and reentrant activation (Spach and Dolber, 1986; Spach et al., 1988; Vigmond et al., 2017; Mahajan et al., 2018).

### Critical Myocardial Mass

A critical myocardial mass is needed for reentry to occur (Garrey, 1914). The low mass of myocytes in the distal PV explains the failure to induce arrhythmias, whereas the larger mass of myocytes in the proximal PV supports the arrhythmogenesis. Moubarak and colleagues described that PV myocardial sleeve are thicker toward the LA than the distal PV in humans (Moubarak et al., 2000), whereas others reported that the PV sleeves are longer in patients with AF than in those without AF (Tagawa et al., 2001; Hassink et al., 2003). Since an anatomical variation in the myocardial thickness of the PV exists between individuals (Nathan and Eliakim, 1966; Hassink et al., 2003), it is likely that a physiologically large myocardial mass in the atrial-PV junction makes one individual more prone to AF even in the absence of structural remodeling than the other.

### Pulmonary Vein Electrogram Morphology

The electrograms in non-remodeled PV contain both atrial and PV components (Spach et al., 1972; Hwang et al., 1999). We observed fractionation in the local unipolar electrograms in the PV which can be explained by the structural separation of the myocytes by collagen and fat tissue (Spach and Dolber, 1986). We reasoned that a larger and steeper activation deflection (B component) reflects the larger myocyte mass in the proximal PV, in which arrhythmias can be induced and a broad wavefront of activation occurs. The small amplitude fractionated signals reflect conduction along the small isolated fibers similar to what can be recorded from infarcted ventricular myocardium (de Bakker et al., 1991). Therefore, the profibrillatory structural properties in the proximal PV can be identified using the morphology of the unipolar electrogram.

### Functional Pulmonary Vein Properties

The atrial-PV junction in the absence of remodeling exhibited local heterogeneity in refractoriness with the shortest values in the proximal PV. This seemingly contrasts findings in non-remodeled PV from guinea pigs and dogs in which shorter action potentials appeared in the distal PV than in the proximal PV (Cheung, 1981; Hocini et al., 2002). It cannot be excluded that recording techniques have influenced what was considered proximal and distal. Nonetheless, heterogeneity in refractoriness in both scenarios is proarrhythmic because unidirectional block with subsequent reentrant activation can occur following a premature beat originating from the tissue with the shorter refractory period (Mines, 1913; Coronel et al., 2009).

The relatively higher diastolic stimulation threshold in the proximal PV than in the distal PV is an expression of decreased excitability and may facilitate local activation block. The decreased local excitability can be caused not only by an intrinsic change in sodium channel density but also by source-to-sink mismatch in this tissue with clearly separated myocardial bundles (Figure 2). Lee and colleagues reported that conduction slowing and block occurred more often in the atrial-PV junction than in the LA or in the distal PV in patients with cardiac disease but without a history of AF (Lee et al., 2012). They did not test site-dependent inducibility of atrial arrhythmias as we did. Others observed reentrant activations in isolated PV preparations by optical mapping (Arora et al., 2003; Po et al., 2005).

### Wavelength

Collagenous septa within the (ventricular) myocardium can cause a tortuous activation path with a normal conduction velocity (0.7 m/s), while an apparent conduction velocity as low as 0.03 m/s can be observed if one considers a linear activation path (de Bakker et al., 1993). If we estimate the reentrant activation wavelength in the proximal PV (refractory period (170 ms) × apparent conduction velocity (0.03 m/s)) to be 5.1 mm, the diameter of the reentrant circuit is about 1.6 mm. Since the width of the proximal PV sleeve myocardium is in average 1.8 mm (1.35 mm myocardium + 36% collagen-fat tissue), the entire reentrant pathway can take place intramurally within the PV sleeve and requires the presence of a sufficient mass of myocardium. Demonstration of reentry by endocardial or epicardial mapping in the PV is, therefore, not feasible even with a high spatial resolution of the mapping array. Optical mapping techniques are used to reconstruct intramural activation pathways in atrial myocardium (Hansen et al., 2015), but the feasibility of this method in the PV wall has not been demonstrated. Therefore, the inference of intramural reentry cannot be tested.

### Atrial Fibrillation Initiation and Maintenance

The triggering properties of isolated PV from healthy animals are disputed (Chen et al., 2000; Hocini et al., 2002; Wang et al., 2003). Wang et al. (2003) reported no automaticity or afterdepolarizations in the PV of 50 dogs, whereas others described automaticity and afterdepolarizations in as many as 71 and 41% of PV, respectively, in a similar experimental setup (Chen et al., 2000). The profibrillatory structural and functional properties of the native atrial-PV junction bring forward the option that the initiation of AF in the PV is based on reentry (de Bakker et al., 2002).

Atrial fibrillation itself leads to infiltration of adipose tissue and extensive collagen accumulation in the atrial myocardium (Ausma et al., 1997; Boldt et al., 2004; Haemers et al., 2017). These structural changes are responsible for an epi-endocardial electrical dissociation that facilitates reentry (Verheule et al., 2014). Reentrant activations are also considered to maintain AF in the atrial myocardium (Moe et al., 1964).

### Clinical Outlook

The PV myocardium that is prone to AF arrhythmogenesis by reentry can be targeted therapeutically by ablation therapy. We speculated that the ablative protocol may be simplified to incomplete proximal PV lesions in patients with paroxysmal AF without structural remodeling and comorbidities. Then, the lesions can target the PV-atrial transition. Future studies can provide insight into the efficacy of such lesions in AF prevention and whether the ablation can be guided by the electrogram morphology (LFE+B) and local gradients in refractoriness and excitability.

### Limitations

Since AF is a disease in adults (Benjamin et al., 1994), we studied adult (+2 years) sheep. Only female sheep were used because male sheep are either killed in young age (<1 year) or exclusively used for breeding. In humans, the occurrence of AF is similar in both males and females (Benjamin et al., 2018). We intended to study the role of the native PV myocardium on the initiation of AF and, therefore, studied healthy sheep without AF remodeling and comorbidities. Comorbidities and remodeling likely contribute to the maintenance of AF.

We reported structural and functional properties conducive to reentry but did not record the reentrant activation circuit. This could not be circumvented by an increased resolution in mapping electrodes because we cannot exclude that the reentrant activations take place intramurally. Others showed using optical mapping that intramural reentrant activations occur during AF in human right atrial preparations (Hansen et al., 2015). The feasibility of intramural mapping in PV has not been demonstrated.

### Conclusion

We observed atrial arrhythmias after premature stimulation of the proximal PV, but not of the distal PV in the absence of remodeling. Distinct structural and functional properties conducive to reentrant arrhythmias with a short wavelength existed in the proximal PV and were identifiable using the typical morphology of the local unipolar electrogram. The initiation of AF in the early paroxysmal AF likely involves reentry in the atrial-PV junctional myocardium.

## Data Availability Statement

The raw data supporting the conclusions of this article will be made available by the authors, without undue reservation.

## Ethics Statement

The animal study was reviewed and approved by the local ethical authorities at University of Bordeaux, France (approval number 7995).

## Author Contributions

LG, CB, SA, VL, MC, MH, LD, and RC performed the material preparation, data collection, and analysis. LG wrote the first draft of the manuscript. All authors contributed to the study conception and design, commented on previous versions of the manuscript, and read and approved the final manuscript.

## Conflict of Interest

The authors declare that the research was conducted in the absence of any commercial or financial relationships that could be construed as a potential conflict of interest.

## Publisher’s Note

All claims expressed in this article are solely those of the authors and do not necessarily represent those of their affiliated organizations, or those of the publisher, the editors and the reviewers. Any product that may be evaluated in this article, or claim that may be made by its manufacturer, is not guaranteed or endorsed by the publisher.
